# How short peptides can disassemble ultra‐stable tau fibrils extracted from Alzheimer’s disease brain by a strain‐relief mechanism

**DOI:** 10.1002/alz.085001

**Published:** 2025-01-09

**Authors:** Ke Hou, Peng Ge, Michael R Sawaya, Joshua L. Dolinsky, Yuan Yang, Yi Xiao Jiang, Liisa Lutter, David Boyer, Xinyi Cheng, Justin Pi, Jeffrey Zhang, Jiahui Lu, Shixin Yang, Zhiheng Yu, Juli Feigon, David S. Eisenberg

**Affiliations:** ^1^ UCLA, Los Angeles, CA USA; ^2^ Janelia Research Campus, Howard Hughes Medical Institute, Ashburn, VA USA

## Abstract

**Background:**

Reducing fibrous aggregates of protein tau is a possible strategy for halting progression of Alzheimer’s disease (AD). Previously we found that in vitro the D‐peptide D‐TLKIVWC fragments tau fibrils from AD brains (AD‐tau) into benign segments, whereas its six‐residue analog D‐TLKIVW cannot. However, the underlying fragmentation mechanism remains unknown, preventing the further development of this type of drug candidate for AD.

**Method:**

To understand the necessity of the cysteine residue of D‐TLKIVWC in fragmenting AD‐tau, we designed a series of peptides of sequence D‐TLKIVWX varying only at the seventh residue, X. To better understand the fragmentation process of AD‐tau, we conducted a time‐course dot blot and EM experiment. We determined the structures of D‐TLKIVWX amyloid‐like fibrils by atomic force microscopy and cryo‐electron microscopy. We studied the complexes of D‐TLKIVWX (X = I, S, R) with AD‐tau by cryo‐electron microscopy and confirmed the binding site between D‐TLKIVWX and Tau through NMR.

**Result:**

These D‐TLKIVWX candidates showed various efficacies in fragmenting AD‐tau in vitro, in which X = Ile was the best performer. From electron microscopy, we discovered that D‐TLKIVWX peptides form amyloid‐like fibrils themselves, and from atomic force microscopy we learned that these fibrils have a right‐handed helical twist, in contrast to the left‐handed helical twist of AD‐tau. From cryo‐EM we learned that D‐TLKIVWX protofilaments bind to tau fibrils of opposing twist.

**Conclusion:**

We find that the amyloid‐like, fibril‐forming property of D‐TLKIVWX contributes to the fragmentation of AD‐tau fibrils. We propose the strain‐relief mechanism of fragmentation and believe the fragmentation of AD‐tau fibrils is driven by the release of torsion in D‐TLKIVWX protofilaments.

